# Basic chloroplast characterizations of *Prunus campanulata* x *kanzakura ‘*praecox*’*, a warm-adapted cherry cultivar in South China (Rosaceae)

**DOI:** 10.1080/23802359.2019.1688109

**Published:** 2019-11-14

**Authors:** Yuzhen Zhou, Bin Chen, Yan Zheng, Ziling Wei, Kai Zhao

**Affiliations:** a College of Landscape Architecture, Fujian Agriculture and Forestry University, Fuzhou, China;; b College of Life Science, Fujian Normal University, Fuzhou, China

**Keywords:** *Prunus campanulata* x kanzakura ‘Praecox’, chloroplast genome, phylogenetics, consanguinity, hybrid cultivars

## Abstract

Flowering cherries are domesticated to cultivars by multiple hybridization from some original species, and there formed various adaptive abilities. Herein, we established the complete chloroplast genome of *Prunus campanulata* x *kanzakura* ‘Praecox’. The chloroplast genome circle (157,917 bp) presented a typical structure of one 85,928 bp LSC, one 19,117 bp SSC region, and two 26,436 bp IRs. It encoded 124 genes, and same conserved tRNA genes and rRNA genes with *P. campanulata*, 37 and 8, respectively. The overall GC content was 36.72%. Phylogenetic analysis confirmed the maternal inheritance relationship that *P. campanulata* x *kanzakura* ‘Praecox’ comes from *Prunus campanulate* with little chloroplast genome change, nested inside subgenus *Cerasus*. This announcement of chloroplast genome would provide helps in further genetic modification and distinction mark study in *Prunus* genus.

Cherries are characterized by beautiful color and large amounts of flowers along with the delicious fruits. There exist hundreds of ornamental cultivars, and they were divided into different application types. In genus of *Cerasus,* original species are heavily crossed in order to strengthen economically and ornamentally characters. The long history of cultivation of flowering cherries, however, has caused significant confusion over names and origins. Thus, we presented the complete chloroplast genome of a hybrid cultivar, *Prunus campanulata* x *kanzakura ‘*Praecox*’*. It owns light red single-five petals, different from parent *Prunus campanulate*. This cultivar is common to south China especially in Fujian, Guangdong that areas with warm climates and acid soil. *P. campanulata* x *kanzakura ‘*Praecox*’* is one of the first flowering prunus, once reported in January of Fujian, near to the flowering time of *Prunus mume*. Around 300 Mb-level genome of *Cerasus x yedoensis* ‘Somei-Yoshino’ (Shirasawa et al. 2019) and *Prunus yedoensis* display inter-specific hybridization between sympatric flowering cherries (Baek et al. 2018), a whole genome level comparison between prunus may be a perfect method in the nearby future to analyze the mechanism of character diversity and provide clear guidance in the multiple-method breeding. Now, high quality complete chloroplast (cp) genome helps differentiate the cultivars and focus on vital functional gene loss or gain in the plastid. Comparative genome of inner or outer species in genus level also provided a new promising method for phylogeny, population dynamics, and species evolution. Thus, it is interesting to assemble and characterize the cp genome of *P. campanulata* x *kanzakura ‘*Praecox*’* to provide a better understanding of the domestication and genetics in this genus.

Plants were located in Fujian province, China (26°20'21.3” N, 113°12'39.6” E), samples for experiments were collected and then preserved in the laboratory of Fujian Agriculture and Forestry University. Total genomic DNA was extracted from fresh leaves by modified CTAB method. The frozen samples including fresh tissues, specimens and sequenced DNA can be acquired by the following voucher specimen accession number (YT-FJ2019-9A, FAFU). PE150 pair-end library strategy were adopted and sequences were obtained by the BGI-500 platform (BGI, Wuhan, China) (Mak et al. 2017). We obtained total about 6 Gb clean reads after removing adapters and low-quality reads by fastp software (Chen et al. 2018) and reads were corrected by bfc, a standalone high-performance error-correcting tool. Then the processed data were extracted and assembled by GetOrganelle v1.5.2 flow, in which core mapping software and assembly tool were bowtie2 and Spades. Random separated reads were then assembled and extended into contigs. Fragments with low sequence coverages were removed as noises during the screening progress by using Bandage v0.8.1 and ultimately formed a complete circle chloroplast. The draft cp genome owned about a 400× coverage, clean reads was mapped to the draft cp genome to check the assembling consistency. The genome was preliminarily annotated for genes and tRNA using GeSeq (Tillich et al. 2017), to adjust the starting position, long repeat sequences were annotated by Geneious. Altogether, we established a length of 157,917 bp complete circle chloroplast genome of cultivar *P. campanulata* x *kanzakura ‘*Praecox*’* with an average GC content of 36.72%. This plastid genome included a length of 85,928 bp large single-copy (LSC) region and a 19,117 bp small single-copy (SSC) region, separated by two conserved 26,436 bp inverted repeat (IRs). The individual parts manifested unbalanced GC contents, respectively, LSC-34.59%, SSC-30.22% and IR-42.53%. After assessment of the assembled plastid genome, we annotated the new cp-genome by online software GeSeq again. We found 124 genes, 37 tRNA and 8 rRNA in the presented circle genome. The intact assembled chloroplast genome of *P. campanulata* x *kanzakura ‘*Praecox*’* and related annotation information can be detected in GenBank (MN537435).

Members in Rosaceae have been reported mingled based on plastid phylogenomics (Zhang et al. 2017). Therefore, we gathered most reported chloroplast genomes in genus Prunus to investigate its hybrid origin. Nearby-species complete cp genomes in Rosaceae were settled and HomBlocks pipeline was adopted to align same blocks along the plastid (Bi et al. 2018). Then, RAxML-HPC program on CIPRES Science Gateway was used to construct the maximum likelihood (ML) tree with 1000 bootstrap replicates as shown in Figure 1. As expected in the total 24 plastids, *P. campanulata* x *kanzakura ‘*Praecox*’* was more closely related to *P. campanulate* and *Prunus pseudocerasus* in the conventional sense of *Cerasus*. This cluster offers a proof that *Cerasus* like *P. campanulate* and its cultivars still own unique positions in the genus *Prunus* in the level of complete chloroplast genome. This is mutually reflected with the traditional taxonomic results. We believe the presentation of *P. campanulata* x *kanzakura ‘*Praecox*’* chloroplast genome would provide vital genomic resources for prunus breeding and fundamental researches.

**Figure 1.**
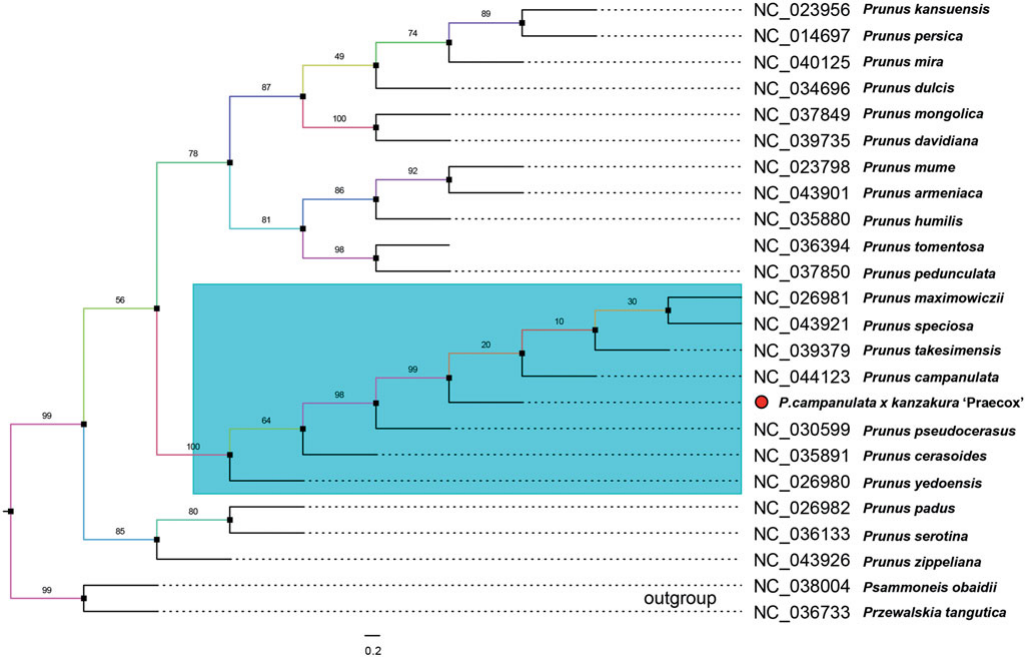
Maximum-likelihood (ML) phylogenetic tree of 24 selected chloroplast sequences with 1000 bootstraps. *Prunus campanulata x kanzakura* ‘Praecox’ were marked with red circle. Genebank accession numbers were listed together with their corresponding species names.
